# Experiences of living without a sense of smell: Like “Being Behind Glass”

**DOI:** 10.1371/journal.pone.0293110

**Published:** 2023-10-19

**Authors:** Lorenzo D. Stafford, Karl Nunkoosing, Mark Haydon-Laurelut, Michael Fisher

**Affiliations:** Department of Psychology, University of Portsmouth, Portsmouth, United Kingdom; Federal Ministry of Agriculture and Rural Development, NIGERIA

## Abstract

This study addresses the paucity of research concerning the subjective experiences of those affected by anosmia. In the study, we interviewed individuals(n = 11) recruited via the charity (Fifth Sense) and used Interpretative Phenomenological Analysis (IPA) to analyse the data. Findings revealed three main themes and seven sub themes. The main themes are Living with Anosmia; Remembrance of things old and new and Resilience. The study reveals the process of becoming aware of being anosmic and the relationships with others in this process including potentially unhelpful minimisations of the impact by professionals. In addition to a sense of isolation and insecurity, living with anosmia for some participants brought with it an identification of being ‘anosmic’ and feeling part of a community. This was in contrast to a general lack of public knowledge and understanding of anosmia. The findings of the study demonstrated the importance of smell to time, place and relationship and the recalling of smells as bringing a sense of connectivity to loved ones, of times past and also a sense of loss of ability. Participants also described the ways in which they coped and adapted to a life with anosmia and focused on positive aspects of life. These findings provide a rich qualitative account of the experience of anosmia. The findings point towards future research which could inform us about the lives of those who are anosmic and currently unaware and of those recently diagnosed, which will create a richer understanding of the experiences of anosmia.

## Introduction

We live in a world of odours and yet we are generally unconscious of this until there is a smell related change in our environment [1]. Moreover, we are generally unaware of the importance of this sense until we experience difficulties. Anosmia, the loss of the capacity to smell, is estimated to affect about 5% of people [2, 3]; it affects more men than women [4]; like sight and vision the sense of olfaction deteriorates with age [5, 6] and its prevalence is higher in black people than in white people [7]. There are a number of diverse causes of anosmia, with the majority of cases due to sinonasal disease (e.g. chronic rhinosinusitis and allergic rhinitis) and post-traumatic olfactory loss and idiopathic cases [8]. One of the symptoms of the current Covid-19 pandemic is the loss of smell and taste functions [9].

Several studies identified safety concerns as a consequence of anosmia [10, 11]. These risks include not detecting gas leaks or smoke, the consumption of spoilt food and the burning of food [10]. However, these practical consequences of anosmia and its threat to health are not as frequently researched as the diagnosis and treatment of the condition.

The personal consequences of anosmia include loss of interest in food, general anhedonia or the inability to feel pleasure, as well as concerns about being exposed to danger because of the olfactory impairment [12]. Anosmia often manifests itself as a perceived loss in the capacity to taste with around two-thirds affected [13], though actual ‘taste’ sensitivity is usually unaffected [14]. This ambiguity results from the fact that being unable to smell reduces our ability to detect the ‘flavour’ of food in our mouth and it is to this aspect that anosmics refer, whereas ‘taste’ is the ability to detect the basic tastants (sweet, bitter, salty, sour, umami) and is generally left intact. The consequences of this are a reduction in the enjoyment of food [15] and a heterogenous pattern in terms of weight gain, with roughly equivalent numbers who gain versus losing weight [16, 17]. In a survey study of 176 patients who used an anosmia clinic, researchers found that changes in weight were associated with dietary factors including changes in cooking behaviours such as increased use of spices to compensate for loss of taste and flavour [18]. More recently, work has shown that weight gain is more prevalent in younger anosmics and accompanied by reports of overeating to derive more pleasure [16, 19], which could be a compensatory response, linked to their reduced flavour detection. This suggests that the reaction of people to their anosmia may well depend on demographics, culture and individual psychological characteristics.

The more general aspects of food cooking and eating are also important to consider, since food is not just about nutrition. We are reminded that whilst all animals eat, only humans cook [20], hence food choice, culinary traditions, the pleasure of cooking and meal sharing contribute to food culture and identity [21]. Cooking and food sharing is also about one’s lifestyle, one’s social relationships and it seems likely that the changes in these aspects as a result of anosmia are key in the reported reduced quality of life [15, 17].

One of the social concerns of individuals who experience anosmia is anxiety about not noticing their own body odour and how others might think that they are malodorous [22]. One consequence of such anxiety is excessive showering and over use of perfumes [23]. This fear can lead to social isolation. Social isolation is also part of managing the hidden stigma of anosmia [24]. Anosmia is an invisible impairment and its presence has to be disclosed, thus the stigma may well be an example of self-stigma. Lillis et al. [24, p.923] describe self-stigma as “… when individuals come to associate themselves with negative characteristics and fear that others will too.” Self-stigma occurs when the person internalises the negative values that society holds about their condition. The possible origin of the stigma of anosmia is in the observation of the tendency of others to either trivialise or catastrophise the anosmia experience accompanied by a disbelief that one cannot smell [12]. These values also contribute to the lack of empathy for the person who experiences anosmia.

Olfaction does not hold the same importance in human environmental awareness as sight and sound, which may contribute to the often-reported lack of empathy for the person who experiences anosmia. Indeed, there are those for whom such olfactory changes go unnoticed. Relevant here is a study of a sample of 8348 participants who self-reported a normal sense of smell; but when subsequently tested, 310 of whom were found to be functionally anosmic [25]. This perceived lack of importance of smell to human existence is reflected in the fact that general medicine does not test the sense of smell during a general medical examination [22, 26]. This disinterest may be due to the current lack of treatment for anosmia. Additionally, anosmics often report a tendency in health care professionals to not take their condition seriously [27].

The majority of studies about anosmia tend to be about the physiology, diagnosis and treatment of the condition. There is a need to further understand the experience of daily life in the presence of the condition. In one of the rare qualitative studies, Erskine and Philpott [26] analysed the personal written accounts of the experiences of anosmia from 71 members of Fifth Sense, a UK charity that supports people who experience smell and taste disorders, and identified several significant negative experiences such as: feeling of isolation, relationship disruption, interruptions of daily life, difficulties in help seeking and health related issues.

The current study features in-depth and reflexive interviews with the participants to enable them to narrate their experiences of living with anosmia. The stance is that the participant is an expert in living with an absent or a compromised sense of smell. Interpretative Phenomenological Analysis (IPA) [28, 29] is employed in order to achieve an understanding of the lives of persons who live with anosmia. The objective is therefore to understand the lived experiences of those individuals living with anosmia.

## Method

### Participants

Eleven individuals (5 women, 6 men, see Table 1) were recruited via Fifth Sense (https://www.fifthsense.org.uk/). An email was sent out from Fifth Sense in 2016 to all members stating that researchers were looking for volunteers for a study examining the experiences of those living with no (or severely impaired) sense of smell and individuals interested in participating were asked to email the lead researcher to arrange a convenient time for the interview. The sample therefore comprised of those who were members of fifth sense who self-identified as suffering from smell loss; there were no other inclusion/exclusion criteria. The study gained ethical approval through the University of Portsmouth’s Science Faculty Ethics committee (Ref: SFEC 2016–038), which complies with the Declaration of Helsinki for Medical Research involving Human Subjects; all participants provided written informed consent.

**Table 1:** Participant demographic and odour test scores.

	Pseudonym	Age	Gender (M/F)	Odour Threshold^a^
P1	Ann	75	F	7.5
P2	Jon	49	M	2.25
P3	Liz	74	F	1.0
P4	Mike	79	M	1.0
P5	Will	55	M	4.75
P6	Ben	58	M	1.0
P7	Paul	60	M	1.0
P8	Julia	63	F	5.50
P9	Sarah	69	F	5.0
P10	Val	77	F	1.0
P11	David	37	M	1.0

^a^Lower figures represent poorer sense of smell. Participants with a threshold of 1 are likely to be anosmic whilst those with scores >1 are likely to be hyposmic. These values can be compared to an undergraduate student sample who completed the same olfactory test (M = 10.0 ± 0.26).

### Procedure

The study took place within the University’s Department of Psychology. On arrival, participants completed an informed consent form followed by an odour smell (threshold) test. Test scores were not part of the eligibility criteria for the study, which was based on participants who self-identified as anosmic and were a member of Fifth sense; however this test provided a method of establishing the level of their odour dysfunction. The odour smell (threshold) determines the lowest concentration of a neutral odour that could be reliably detected. For this test, we used a neutral odour (N-butanol) which is the standard odour for the threshold tests used in this research area [30, 31]. The odourant was prepared using sixteen 250ml squeeze bottles, in 16 dilution steps, starting at 0.125% (Step 1) with each successive step diluted by a factor of two using serial dilution to the lowest (Step 16) dilution. In addition to the odour containing bottles, for each dilution step, two ‘blank’ squeeze bottles (containing dilutant only) were used in the threshold test. Testing commenced by asking participants to smell the bottle with the highest concentration to familiarise themselves with the target odour. They were then presented with the triplet containing the weakest concentration. Following presentation of the last bottle of the triplet (counterbalanced), participants were asked which bottle contained the odour (1, 2 or 3). If the participant answered correctly (and it was the lowest concentration), they were presented with the same triplet again (in a different order) and the task repeated until they made a mistake, which resulted in the triplet containing the next concentration step being presented. Using a single up-down staircase system (as used widely in olfactory research, e.g. [30]), this was then repeated until there were seven ‘turning points’, with the mean of the last four points determining the threshold for the individual. Each bottle was held under the participant’s nose (≈ 2cm) and gently waved between each nostril to ensure optimal inhalation. A blindfold was used by the participants to avoid odour identification. The experimenter wore cotton gloves (Boots the Chemist,) to reduce any cross contamination of odours. Test results and demographic information is in Table 1 below.

#### Sample characteristics

The results of the odour threshold test confirmed substantial loss of smell function (Table 1), though it is evident that some participants (especially ‘Ann’) had some odour function).

Participants were then shown to a different room where they took part in a semi-structured interview.

The Semi-structured interviews were guided by an interview schedule which prompted the interviewers to ask about events that led to the loss of participants sense of smell, reaction to diagnoses, adjustment to their life as a result of olfactory loss, sensory compensation (i.e. do they find other senses are more/less acute), advantages and disadvantages of living with olfactory loss and advice to any individual newly diagnosed with olfactory loss. Semi Structured interviews facilitated interviewers to have the freedom to explore the experiences of participants with the interview schedule as a guide. Interviews were conducted by one of two experienced Researchers (R1/R2): one of whom (R1) had extensive experience in interviews. To provide some consistency, R2 observed two interviews conducted by R1 and these observations were then reflected on by R1 and R2. All interviews were conducted face-to-face in a quiet office at the University. Interviews were digitally recorded and transcribed verbatim. In this article, extracts are identified with researcher-assigned participant pseudonyms to preserve anonymity.

#### Data analysis

The audio recordings of the interviews were transcribed verbatim in Microsoft Word by the Research Assistant (MF). Each transcript was checked against the audio recording to ensure that it was a faithful textual representation of the interview. The inductive and idiographic analysis [32] of the transcripts was guided by IPA’s concern with the participants’ experiences and the meanings that they attached to these [29]. One of the concerns of IPA is the notion of the ‘double hermeneutics’, that is we as researchers sought to make sense or interpret the participants’ interpretations of their lived personal and social experiences of anosmia. Researching with IPA is about two voices, that of the participants and those of the researchers. A stance of openness to the unusual permeates all stages of the analysis. We also distinguish between analysis and interpretation. Analysis is about immersing oneself in the data to seek out shards of information, events, experiences that can potentially contribute to our knowledge of living without the sense of smell. Whilst interpretation is the intellectual act of seeking the meanings of the particular experience that we have identified as being of interest to the phenomenology of anosmia.

The analysis began with the 3 researchers independently reading each transcript a number of times. The researchers made notes of items and issues that are potentially informative of the phenomena of living with anosmia. We engaged in line by line coding of the transcript and used the ‘New Comment’ facility in the ‘Review’ procedure of Microsoft Word to write these observations in the right-hand margin of the line numbered transcripts.

The next stage of analysis and interpretation was conducted via an online video platform and in person with the first 3 authors, after the annotated transcript or tables had been shared. We compared and discussed our analyses. Attention was paid to the following: How experiences and their contexts were described and interpreted by the participant; the language used; concepts that are relevant to the experiences of anosmia; to the personal, social and relational implications of anosmia. The researchers engaged in reflexive conversations concerning their various theoretical and methodological orientations, including critical disability studies, systems theory and knowledge of the psychological impact of sensory impairments. These conversations significantly contributed to the quality of the interpretations. Sessions identified themes that were particular to the participant under consideration. The process continued until we reached a state of saturation, where no new insight was present. In each of these sessions we considered between 2 to3 transcripts. We continued with this process until all the transcripts had been analysed and themes common to all the transcripts had been identified. One of the researchers re-read the transcripts, and the associated notes and themes produced during the earlier stages of analysis, to produce the subthemes and superordinate themes. A final meeting and discussion of all the researchers sought to identify the final themes that are presented here (Table 2).

**Table 2:** Themes and subthemes.

Themes	Subthemes
1. Living with Anosmia	1. On Becoming Anosmic
	2. Anosmic Identity
	3. **Trivialization of anosmia by health professionals**
	4. Quality of Life
2. Remembrance of things old and new	1. Odour Memory
	2. Food Odours
	3. Odour and New Memories
3. Resilience	

### Findings

There are three main themes: 1) Living with Anosmia, 2) Remembrance of things old and new, and 3) Resilience. The first of these refer to narratives about embracing the anosmic status and the subsequent development of an ‘anosmic identity’. Since all the participants have a past when they had intact olfactory capacities, they rely on these memories to make sense of their current world of limited smells. They also rely on these memories to create new sense of their world without smell. These main themes are also accompanied, for the most part, by subthemes (Table 2).

### Living with anosmia

Participants described living with anosmia including the process of becoming aware of their anosmia. This was experienced over varying time frames and with varying degrees of personal salience and identification. Health Care Professionals were experienced by some participants as being unhelpful, in terms of minimising the significance of anosmia. Living with anosmia also affected the quality of life of some participants, bringing with it experiences of isolation, insecurity and feeling cut off/disconnected from loved ones. Other participants did not experience anosmia as so significant in their lives.

#### On becoming anosmic

The onset of the anosmia and the awareness of the inability to smell differs among the participants. Ann whose anosmia was caused by a head injury, became aware she could not smell eighteen months after the injury, when her husband came into their house and remarked that the house was:

“Absolutely full of gas. And he (the husband) says ‘what are you doing?’

I say: ‘Nothing.’

He said: “But can’t you smell that gas?”

For other participants, the awareness of anosmia was sooner. Mike who had dived into the sea hitting his head on the sea bed recalled that either the same or the next evening he awoke from being asleep to a distinctive odour:

“Most distinctively the smell of wood burning. (I) looked around the house, couldn’t see anything”.

From this experience, Mike deduced that there was something wrong with his olfactory functions which he attributed to the diving mishap.

Paul became aware that his sense of smell had been affected within a week of an accident that led to the occurrence of the anosmia. He then proceeded to test his sense of smell:

“…as I started to put things together, I realized that something wasn’t quite right, with the smell…. did a few tests at home with a cup of bleach and a cup of petrol and a cup of water, and seeing if I could, and realized that there was no difference…”

These findings are in accord with other work that shows considerable variety in people’s unawareness of their anosmia [25]. This also reflects our generally poor awareness of our sense of smell and our subjective estimation of our sense of odour function is inaccurate when compared to the results of objective testing of our olfaction [2].

#### Anosmic identity

For some participants, identifying with being ‘anosmic’ and the feeling of being part of a community was important. This is especially so, given the low exposure of anosmia in the media compared to other sense related conditions and a general lack of public knowledge and understanding about the condition. Val, who has been anosmic for most of her life, recently listened to a radio programme on the topic that cheered her and encouraged her to join ‘Fifth Sense’:

“…enormously cheering, that somebody was finally addressing the problem, because there are a lot of us around”.

“…to sign up to this society [Fifth Sense] that’s trying to promote it … And I thought, well good on you mates, somebody’s doing something about it”.

Similarly, Jon talked about the importance of being part of the (Fifth Sense) community:

“So what I got out of Fifth Sense was the understanding was that there was a significant amount of people that this (anosmia) affected……. That I was interested enough to turn around and commit myself to that, which would show other people.”

For Liz, the anosmia is a hidden stigma that she manages by not revealing the condition to those who do not need to know, in contrast to her membership of 5^th^ Sense where she meets with others who experience the same condition.

“… it’s not a particularly nice thing to have in common. It’s a negative, not a positive. I just went on holiday with a group of people I didn’t know at all from not far from me, and from that group I made some really good friends. People I really liked. None of them knew that I’m anosmic. I didn’t tell them, because it’s not something that I share, but people I’ll be seeing again, because I just like them and we got on.”

By not disclosing her anosmia, Liz also saves herself the bother of having to explain the condition to other people. In this sense, Liz is also engaging in the process of ‘self-stigma’. Self-stigma in this context is the internalization of what is perceived to be negative attitudes of the public towards people with anosmia. Hence, as seen in other domains [24], it could be that she fears some negative reaction to telling others of her inability to smell. Anosmia is a hidden impairment and one way to manage its stigmatizing potential is simply not to disclose that one lives in a world without scent.

#### Trivialization of anosmia by health professionals

Some participants talked about the attitude of medical practitioners in appearing to ‘downplay’ the importance of the sense of smell to one’s wellbeing. Will was recovering in hospital from a stroke and realized he could not smell, he described the response from staff:

“Nobody seemed to want to address the situation, and I did speak to my neurosurgeon, just an informal chat. He said what about things, and I said, I can’t smell or taste anything, and his reply was, ‘don’t buy any good wine’. Well that summed it up. In other words, nobody really wanted to know anything about it.”

David, who had additional health conditions, stated his dissatisfaction with the attitude from a physician who said:

“…’it’s just your sense of smell, it’s not important’, but for me, it’s equally important” (David).

Participants experiences of encounters with health services are consistent with existing literature demonstrating that, in general, medical professionals do not view anosmia as a significant health issue [26]. Relevant here is the work of Reeve [33] who proposed that our sense of identity is constructed in our interaction with others and this can sometimes cause a conflict in self-identity. For those participants who experienced anosmia as a significant loss there was an incongruity with the views and interactions with medical staff, inducing a conflict in how they view themselves.

#### Quality of life

Anosmia is a hidden impairment that nevertheless can have adverse consequences to one’s quality of life of which personal relationships are an important part. Participants often contrasted the loss of their sense of smell to the loss of other senses, particularly to hearing and sight as David describes:

“But nearly everybody forgets that I can’t smell. Everybody! Even my wife sometimes. Because it’s not like not seeing. You’d be able to tell if somebody’s blind.., but with sense of smell, people just forget about it”.

Liz described a hierarchy of impairment [33]. Liz described being silenced by this hierarchy, she does not speak about her experience of anosmia as she anticipates the response.

“Well, if it’s vision or hearing, it’s very obvious, and there’s lots of aid for it, but for taste and smell, there’s neither sympathy nor aid available. It’s not understood, and it’s not recognized by other people. If I’m with people I don’t know, and we’re eating together, I don’t tell them….. you feel deprived of something that we accept as norm, in the western world, we accept a normal part of life as eating out with friends and that experience…. It is a disability, without a doubt”.

For Julia, it was the loss of enjoyment of food itself that led to the exclusion of food as a topic of conversation.

“I don’t tend to talk about food much because it’s not a pleasure for me, I suppose, so that aspect of my life has gone.” (Julia).

For other participants, the loss of connection and empathy were particularly palpable in their inability to smell the unique odour of loved ones. Paul describes the impact of anosmia in the connection he feels with his children:

“My children are adults now, but they still smell like my children, but every time I see them and I give them a hug, I don’t get that connection that I used to”.

Paul paints of picture of a relationship with his children being frozen in time and of disconnection. Paul goes on to say:

“I sometimes feel if I’m in a room, I’m sort of behind a barrier. Behind a glass, so to speak, and I feel a little bit like that emotionally with people sometimes. I know they’re there, and it’s someone I like or love, but there is some connection that’s missing”.

Pauls use of imagery (‘in a room’, ‘behind a barrier’, behind a glass’) both highlights experiences of isolation and adds to the sense of invisibility described above by Liz and David. Of note here is how anosmia constitutes an invisible barrier that exerts negative influences about Paul’s intimate relationships. The absence of a sense of smell in one of the partners in a relationship requires both partners to adjust to this impairment. Paul’s emotional distance could have adverse effects on his relationships; an adversity that is mitigated when the other party understands and adjusts to Paul’s apparent emotional distance. Anosmia does not simply affect the man or woman with the condition; it affects other persons in the anosmic person’s social network. The degree of adjustments required is proportional to the closeness of the relationship.

Julia also adds to this in her description of the reduction in emotionality (the use of the word ‘blank’) as touch remains alone without smell:

“But having a hug with someone and being able to smell their smell is very reassuring, I can’t do that. My husband always used to smell a certain way, but he doesn’t smell of anything now. It’s just completely blank. So that is quite difficult…”

Similarly, David highlights the emotionality of the ability to smell:

“There’s a bit less of a connection between us, and it’s not just sexual relations, it’s that security that your partner gives you, and part of the security is how they smell, and being familiarized with that smell of the person. So yes, nothing’s replaced that”.

Jon’s sense of loss of connection and the impact of anomia on his life took longer to develop. Jon describes the situation of disclosing to a stranger in a work setting that they could not smell who replied:

“Wow, you can’t smell. That is a really big deal”

This caused Jon to reflect:

“And I’m like, it never really occurred to me- I’d never really looked at it like that. I just thought it was one of those faculties that I’ve done without, and there’s a lot of worse faculties to lose”.

Another’s perception of the significance of anosmia led Jon to consider further the meaning of this for him and in recent years, Jon started to think more about his loss of smell. Jon describes wanting to talk with others about anosmia:

“It’s really affected me. It’s affected me enough to want to give up half a day and come down (for this interview), because I wanted to talk about it, because no one has asked me about it..…not even my wife”.

It is important however, to acknowledge that for some individuals, losing their sense of smell did *not* appear to influence their quality of life substantially. For Ann:

“I wouldn’t say that it’s impacted on my life greatly…. I do like the smell of roses, and I do like perfume, but I wouldn’t say psychologically it affects me”.

For Sarah her ability to smell had deteriorated gradually over the previous 6 years and apart from disappointment in not being able to appreciate wine, she, like Ann also found Anosmia to be less significant an issue:

“So generally, it doesn’t bother me. I don’t get upset about it, but I’m not the sort that does, I suppose”.

Sarah also described her relationship to food more generally further highlighting that anosmia was not a significant issue for her due to her lack of interest in food:

“I’m not interested in food. It’s a means for meeting people. When we go out, the food’s extra. Beside the point, it’s going and socializing which is more important than the food. I’ve never been a foodie” (Sarah).

### Remembrance of things old and new

Smell evoked place and time and relationship. There was both some comfort in reminiscence of smell (as both embracing of oneself and as providing connectivity to loved ones and times past) as well as a sense of the loss of an ability. Memories of food odours in particular offer a kind of reverse of the Proustian moment where memories provide context and meaning to the experience of cooking and eating with anosmia in the present.

#### Odour memory

For all participants recollected odours were associated with intense memories. Val recollected a memory many years previous of porridge (oatmeal) cooking overnight for hours, its ‘peaty’ scent and the poignant effect it had upon her as a child:

“Well, I was very young….Just feeling wrapped around in a warm embrace. You were warm, you were secure. Any minute there was going to be food in your belly. I mean, what a great feeling is that? Great, great feeling” (Val).

Val expresses the enveloping corporeal nature of the childhood experience, being held in its ‘warm embrace’ along with the anticipation of eating and how much this experience and the memory of it means to her. An important aspect of that embodied experience is the smell of food and the anticipation of breakfast.

Mike recalls the experience of the evocative and sensual smell of perfume. Smell, as for Val, is embodied and for Mike speaks both to his youthful attendance at a ‘dance’ as well as the present:

Mike’s olfactory memory incorporate courtship rituals of his youth involving the sensual smell of perfume on the female body. In his present life, as a gesture of solidarity, his wife does not wear perfume.

“When I was a young chap, and go to a dance and you meet a nice girl and you dance with her, and you smell the perfume and think, that’s nice. And my wife doesn’t wear perfume anymore, because I can’t appreciate it, and I did like that”.

Julia describes her experience of her father’s greenhouse and of visiting one following losing her ability to smell:

Julia associates memories of her father with place and smell, of her father in his greenhouse and the evocative smell of fragrant herbs, flowers and vegetables growing.

“I got a greenhouse, and my memory of my father is in a greenhouse, with all the smells that go with it. I mean, I can imagine what they smell like in my head, but I can’t actually smell them. And I half expected it to be a bit like that [i.e. when she smelled the odours in the greenhouse], but it wasn’t. Nothing like that. I couldn’t pick up any. It seems as if you’re feeling for something in the dark” (Julia).

A compromised sense of vision as ‘feeling for something in the dark’ is invoked as an analogy for evoking a fragrance that is present but is inaccessible because of anosmia.

Like Val and Mike, Julia’s memories of smells and the places, relationships and times they evoke persist. Julia attempts to recreate the smells by an act of the imagination and of recollection. However, there is a gap in Julia’s experiences of imagining the smells of the greenhouse ‘in my head’ and the smell as directly experienced and in that gap Julia, using a visual metaphor of the dark, is searching, feeling her way for the smells she connects with the memory of her father. Smell plays its part in the reminiscence of odour-associated memories providing some comfort in reliving those times juxtaposed with the loss of ability to live those experiences in the present.

#### Odour memory-food

The social aspects of cooking and eating are often overlooked in anosmia research which tends to focus on the sensorial loss. The uniquely human act of food preparation and cooking [20] is essentially about ‘giving’ of one’s time to someone (including oneself). Cooking and eating reflect our own cultural and individual history [21]. Across our participants, we found a complex, interweaving pattern of these factors, perhaps like a ‘phantom limb’ phenomenon (but not ‘phantosmia’) [e.g. 34], with a memory of how a food use to smell (before anosmia), its socio-cultural association (esp cooking) and how the food is now represented in memory. For instance, for some participants, the habitual use of certain food ingredients, which although no longer detectable by their smell were nevertheless still much prized:

“I suppose individual items I couldn’t live without would be onions and garlic. They’re terribly important” (Val).

Hence, it is perhaps the ritualistic use of these ingredients that were essential to Val and not simply her ability to detect their odours. The feeling of ‘imagining’ a smell in one’s mind was referred to by many participants with reference to food and chimes with the analogy of a ‘phantom limb sensation’ (PLS). PLS is a phenomenon experienced after limb amputation with several neurological theories to explain its mechanism [34]. The similarity with anosmia is that some participants in this study also have food memories that are evoked in the absence of the olfactory component of flavour:

“If I ate an apple, although I couldn’t taste it, I could remember what an apple tasted like, so I would still put two and two together that I’m eating an apple. But after a while, I’m thinking, exactly how does an apple taste? What does an apple taste like now?” (Paul).

For Liz, who related her experience of eating crisps, the phantom sensation was even stronger,

“There’s something in the flavour (of a potato crisp/chip) that I’m picking up… I wouldn’t be able to tell you whether it was salt or vinegar or cheese and onion, but I can tell it’s not ready salted. It’s more interesting than that” (Liz).

It therefore appears that although those with anosmia could not detect the flavour (combined smell and taste of food), other strategies sometimes gave them ‘some’ perspective, such as mouth feel, or the texture and temperature of the food they were eating.

The interplay of socio-cultural and sensory factors was also evident in participants’ shared experiences of dining. For Mike, the taste (flavour) of Indian food was much better when prepared by his son-in law, who used lots of his own herbs compared to the dishes of Indian restaurants which was “just a hot taste”. As the experience of his son-in-law’s food would have been after he lost his sense of smell, this suggests that the differences he perceived are unlikely to be due to perceived sensory differences. Perhaps here, it was the social act of watching the food being prepared by someone close, together with the visual presentation that contributed to the differences in flavour sensation and ultimately enjoyment.

Similarly, for others it was the ritual of certain meals and the rich positive histories that still made them want to cook and enjoy that same food. Jon spoke of the traditional British family tradition of the Sunday roasts:

“I do know though that if we’re going out to lunch, and it’s a Sunday roast, I’m like, yes! But that’s not the smell of Sunday roast, that’s just because Sunday roast was always the best meal of the week. When you were a kid, it was the constant, and when you were a teenager and all you did was eat rubbish food and drink too much and not get enough sleep, it was like, oh man! I’m going to eat some proper food, and you know you were going to eat some proper food, so yea. I think it’s a bit more than a smell, I guess is what I’m saying. There’s a connotation attached to it isn’t there? It always felt good”.

The above accounts emphasise the importance of individual experience of food for those with anosmia and how rituals and previous eating and food related experiences provide meaning and context to the experiences of cooking and eating now. The idea of Proustian memory where odours evoke long lost memories is experienced in reverse for some individuals, where the memory of a previous episode triggers intense feelings, perhaps ‘colouring in’ (i.e. compensating) for sensorial loss.

#### Odour and new memories

Some participants described how the inability to smell meant that the acquisition of new odours is no longer possible. During one of the interviews, the interviewer was talking about his own love of food and what he might prepare to eat that evening (pesto with fresh basil):

“I’m trying to emphasize for somebody who loves food not being able to smell it” (Interviewer).

“Sorry I can’t smell it” (P10, Female).

“So you don’t know what basil smells like?” (Interviewer). “No” (P10, Female).

Hence, it was not just that the participant could not recreate the odour in her mind, but it is almost certainly the case, she had never smelled basil in her life, since she lost her sense of smell when young and at a time when fresh basil was not widely either used or available in the UK. This was echoed by another participant who reflected about how it would feel if she had her sense of smell restored and relating to her stored memory:

“I would know it was a strawberry by smelling it. So[since] I remember that smell.

Some smells I wouldn’t know, because I’ve never tasted them. Maybe some fruits and vegetables that weren’t available in this country when I could smell (Liz)”.

In one of the interviews, the participant talked about his recollection of odours associated to seasons (e.g. Autumn) and what it would be like if he could smell those odours now:

Paul: I think if I was to smell it again, now, obviously I would recognize it immediately. So although I say I’ve forgotten stuff, I think I’ve forgotten how to recall that smell.

Interviewer: So you’re not creating a memory of smell, because you’re experiencing new smells, novel smells, so you can’t create-

Paul: “No, I guess I’m not adding to what I know”.

Reflecting on these accounts, it seems probable that any new experiences for individuals after losing their sense of smell would likely be qualitatively impoverished due to the loss of contextual (i.e. odour) cues. Consequently, the recollection of those experiences would also be somewhat reduced which is consistent with work showing those that have a poorer sense of smell, also have poorer memory recall [35].

### Resilience

All participants were keen to point out that they were still able to live relatively full lives. Participants highlighted their adaptation to anosmia and in particular avoiding paying excessive attention to anosmia, thinking comparatively about other possible and actual difficulties in their lives and the importance of focusing on positive aspects of life. When asked what advice he would give to a wine lover who has loss their sense of smell, Jon who still enjoys drinking wine replied:

“I’d say, “Man up!” [laughter] No, I would say, “Look, I’ve lived for a significant- or lack of ability to smell. But I’ve enjoyed some fine wine, and I continue to enjoy fine wine. And I find other ways to enjoy it”.

Similarly, Liz, when asked about what the future held for her, responded:

“Well you have to make the best, and I try not to let it dominate. As I said, my tactic’s been to not think about it too much, just to try and get on”.

Mikes recollection of being able to smell many years ago did evoke sadness balanced with a sense of acceptance:

“The lovely smell of the brewery. It’s good to be alive. I can’t do that anymore. I miss it. I do miss it. I still miss it. I mean, I love flowers, I love the country side, and I do miss that. But, you know, with a lot worse things, one accepts it. I’ve got many other things to be grateful for” (Mike).

For Will, the life-threatening nature of the event that caused his anosmia helped to keep a sense of proportion despite his experience of anosmia as ‘very debilitating’:

“I’m lucky to be here. I mean, I ended up receiving ten-minutes of heart massage to get me going again. So I’m lucky to be here, count your blessings. Okay you’ve lost a couple of your senses [smell and appreciation of taste/flavour], but it’s not the end of the world. It’s very debilitating. As I say, many disadvantages to it, no advantages” (Will).

Ben’s advice to new anosmics was to:

“..find strategies for making that work for you and I would give the example of me not dwelling on it, but trying to find ways of compensating for it, like if there are things that you can detect, making the most use of it. So, if you’ve got a sweet tooth, go for it, enjoy the sweet things. And again, yea, go to the gym and work it off” (Ben).

Like Liz, Mike highlights the importance of not spending too much time ‘dwelling’ on anosmia (Liz noted her tactic above of ‘not think about it too much’). Julia offered her tips relating to food and odours:

“…to keep that memory of what things used to taste like. Try and remember what they taste like, and that’ll give you more pleasure whilst eating at least” (Julia).

Sarah’s advice was:

“I wouldn’t get into a state about it. It’s just annoying more than anything. Sort of maybe you’d talk to someone about it, but it depends on how old you are. You know, where you are in life. How it would affect you. So, don’t take it to heart too much. There’s worse things”.

Sarah’s description shows how she manages anosmia and where she places it in her life (‘annoying’). For Sarah, how anosmia impacts a person also depends on one’s age. In congruence with other participants here, this shows how thinking comparatively (‘there’s worse things’) may in her view support a life with anosmia.

## Conclusion

This qualitative study explored experiences of people living with anosmia. To date, most of the studies in this area have examined anosmia as a medical issue and focused on the consequences of the loss of odour function [e.g. 12, 13]. In depth interviews and Interpretative Phenomenological Analysis (IPA) allowed for an analysis of rich interview data contributing to knowledge of experiences of anosmia. Three main and seven sub themes were produced. The theme of ‘living with anosmia’ highlighted the process of becoming aware of being anosmic, of professional’s unhelpful minimisation of anosmia and of anosmia’s impact on participants quality of life. Although able to live relatively full lives, there were also experiences of a sense of isolation, of insecurity and of feeling disconnected from loved ones. However, living with anosmia, at least for some participants, included identifying with being ‘anosmic’ and feeling part of a community. This was a resource in the context of a general lack of public knowledge and understanding of anosmia. The theme of ‘Remembrance of things old and new’ highlighted the evocative role of smell, suggestive of place, time and relationship. The reminiscence of a smell might bring an experience of connectivity to loved ones and times past and in doing so be of comfort as well as bring forth a sense of loss. The third main theme ‘Resilience’ showed how participants described the ways in which they coped with anosmia and managed to live meaningful lives. Participants’ adaptation to anosmia included avoiding paying it excessive attention, focusing instead upon other possible and actual difficulties in their lives as well as the positive aspects of life. This study showed the willingness of participants to speak and consider the meaning of anosmia in their lives and to share and make sense of their experiences.

Further research is suggested in a number of areas and in particular, the impact of Health care professionals. As evidenced in the work here and elsewhere [27], there is a real need for better informed professional staff (including general practitioners) and research that explores the attitudes and actions of medical and allied professionals concerning anosmia may in turn support the development of effective training initiatives. We suggest that such training should go beyond the facts of functional impairment and highlight the potential psychosocial implications of anosmia and the impact of the trivialisation of this condition by professional staff. Additionally, given the heightened risk of mental health problems (including depression) in anosmic populations, training should also highlight this aspect to ensure healthcare staff are more sensitive to its prevalence [36].

### Limitations

This study utilised a purposive sample of members of the charity for anosmia, Fifth Sense. However, members may possess a shared narrative or common set of experiences that others who are not members of such an organisation might not share. Future work could reach out to those individuals who have not taken the step to join such an organisation. Additionally, the study did not focus on the experiences of particular age-related cohorts or professional cohorts and it would seem likely that concerns and experiences would be different in other cohorts including and not limited to gender and age. Moreover, whilst the age range in the current study is consistent with previous research and typical of this condition (e.g. mean age 55yrs, [16]; 31-80yrs, [26]; 56yrs, [34]), this may change in the post Covid19 era and is something to consider for future research. Interviews/focus groups with younger anosmics would also be beneficial to further elucidate the age related differences in weight gain [16, 19]. We also acknowledge that the current sample did not comprise of individuals with ‘congenital’ anosmia. Finally, it is noted that since interviewing took place face to face at a university campus, this limited participation for those unable to travel; Online and different interview sites would have expanded the number of potential participants.
